# DRAWING AS A METHOD TO DETECT SOCIO-COGNITIVE PERCEPTIONS OF HUMAN LONGEVITY IN EARLY CHILDHOOD

**DOI:** 10.1093/geroni/igad104.0788

**Published:** 2023-12-21

**Authors:** Alexander Bishop, Larissa Callaway-Cole, Hailey Grimmett

**Affiliations:** Oklahoma State University, Stillwater, Oklahoma, United States; Oklahoma State University, Stillwater, Oklahoma, United States; Oklahoma State University, Stillwater, Oklahoma, United States

## Abstract

The aim of this investigation was to understand how young children socially and cognitively perceive human longevity through drawing. Data for this study were collected from young children, ages 3 to 6, enrolled in Pre-K and Kindergarten classrooms through a university-supported early childhood education center. Investigators first conducted brief open-ended interviews to gauge participant understanding of the concept of being “old.” Participants were then asked to draw a self-portrait image of what might look like at 100 years of age. Finally, participants were asked to describe their picture as well as share a story regarding the self-portrait image they had crafted. Images were decoded and analyzed relative to depicted content, pattern and position, and color preferences. Findings highlight reliance on internal working models which appear to highlight socially emergent and learned attitudes, beliefs, and stereotypes about growing old. Further discussion relative to underlying indication of emotional development will be highlighted.

